# Modernising antiviral drug discovery: harnessing medicinal plants through machine learning and metabolomics to target the SARS-CoV-2 main protease

**DOI:** 10.1007/s40203-026-00620-9

**Published:** 2026-04-17

**Authors:** Anathi Msobo, Pfano Witness Maphari, Gerrit Koorsen, Abdel Nasser B. Singab, Msizi I. Mhlongo

**Affiliations:** 1 Imbewu Metabolomics Research, Department of Biochemistry, Faculty of Science, University of Johannesburg, Auckland Park, Gauteng 2006 South Africa; 2 Research Centre for Plant Metabolomics, Faculty of Science, University of Johannesburg, Auckland Park, 2006 South Africa; 3 Department of Biochemistry, Faculty of Science, University of Johannesburg, Auckland Park, Gauteng 2006 South Africa; 4 Department of Pharmacognosy, Faculty of Pharmacy, Ain Shams University, Cairo, Egypt; 5 Centre for Drug Discovery Research and Development, Faculty of Pharmacy, Ain Shams University, Cairo, Egypt

**Keywords:** COVID-19, Main protease, Medicinal plants, Machine learning, Metabolomics

## Abstract

The COVID-19 pandemic highlighted critical limitations in the speed, scalability and translational efficiency of conventional antiviral drug discovery. Although vaccines and repurposed antivirals have reduced disease severity, the continued emergence of SARS-CoV-2 variants and breakthrough infections underscores the need for sustained discovery of novel therapeutics. The main protease (Mpro), an essential and highly conserved enzyme required for viral replication remains a validated and attractive antiviral target. Medicinal plants represent a vast and underexplored source of structurally diverse bioactive compounds with antiviral potential; however, traditional plant-based drug discovery approaches are often constrained by reliance on ethnobotanical knowledge and fragmented screening workflows. This review critically examines emerging strategies that integrate machine learning, LC–MS-based metabolomics and network pharmacology to modernise medicinal plant-based antiviral discovery. We highlight how machine learning enables data-driven prioritisation of candidate compounds and plant species beyond well-studied taxa, while metabolomics provides experimental validation through comprehensive chemical profiling and dereplication. Molecular docking and molecular dynamics further refine candidate selection by evaluating binding modes and stability, whereas network pharmacology offers systems-level insight into multitarget and multipathway effects. Importantly, we discuss key limitations of these approaches, including data bias, model interpretability, and gaps between in silico prediction and experimental validation. By synthesising these methodologies into a unified computational-experimental pipeline, this review provides a critical framework for accelerating the discovery of plant-derived Mpro inhibitors and supports the development of resilient antiviral strategies for current and future pandemics.

## COVID-19: overview and rationale for continued antiviral discovery

COVID-19, caused by severe acute respiratory syndrome coronavirus 2 (SARS-CoV-2), emerged in late 2019 and rapidly evolved into a global health crisis. The virus is an enveloped, positive-sense single-stranded RNA coronavirus characterised by efficient human-to-human transmission and a wide clinical spectrum ranging from mild respiratory symptoms to severe pneumonia, acute respiratory distress syndrome and multi-organ failure (Zhou et al. 2020a, b; Amat-Santos et al. 2020; Liu et al. 2024; Rizwan et al. 2020). Global efforts led to unprecedentedly rapid development of vaccines and antiviral therapies, including mRNA vaccines and direct-acting antivirals such as Remdesivir, Paxlovid, and Molnupiravir which substantially reduced hospitalisation and mortality rates (Polack et al. 2020; Tang et al. 2022; Extance 2022).

**Fig. 1:**
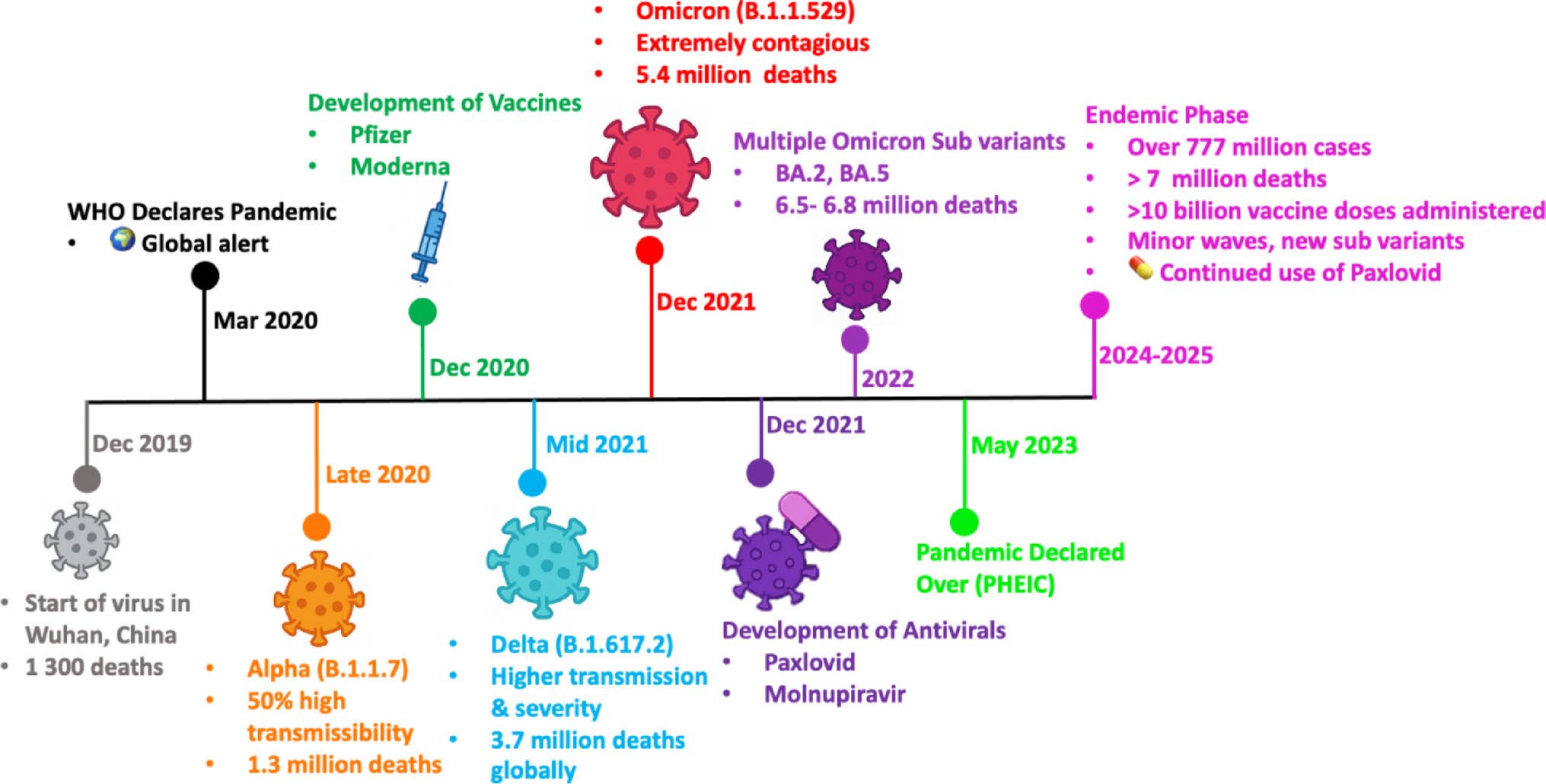
Timeline of major events and milestones during the COVID-19 pandemic (2019–2025). The figure outlines key phases of the COVID-19 pandemic, beginning with the initial outbreak in Wuhan, China (Dec 2019), where over 1300 deaths were reported. It highlights the emergence of major SARS-CoV-2 variants Alpha (late 2020), Delta (mid-2021), and Omicron (Dec 2021) each associated with increased transmissibility and global mortality. The timeline marks the development and rollout of the first mRNA vaccines (Pfizer-BioNTech and Moderna) in December 2020 and the subsequent approval of oral antiviral drugs (Paxlovid and Molnupiravir) in December 2021. By 2022, multiple Omicron subvariants (BA.2, BA.5) were circulating, contributing to further deaths. In May 2023, the World Health Organization (WHO) declared the end of the Public Health Emergency of International Concern (PHEIC). Entering the endemic phase (2024–2025), SARS-CoV-2 continues to circulate with minor waves and emerging subvariants. To date, over 775 million cases, more than 7 million deaths, and over 10 billion vaccine doses have been reported worldwide. Continued use of antivirals like Paxlovid remains essential in managing the disease during the endemic phase

Despite these advances, SARS-CoV-2 continues to circulate globally, with the emergence of variants displaying increased transmissibility and partial immune escape (Tao et al. 2021; Thomson and Willett 2022). In 2023, the World Health Organisation declared the end of the Public Health Emergency of International Concern with recurrent waves and evolving subvariants (WHO 2025). This ongoing circulation, coupled with the virus’s capacity for mutation, highlights the limitations of reliance on vaccines and a narrow antiviral arsenal alone. Consequently, there remains a clear need for continued antiviral drug discovery, particularly targeting conserved viral proteins that are less susceptible to mutational escape. Among these, the SARS-CoV-2 main protease (Mpro) has emerged as a central focus for next-generation antiviral development. These stages of viral entry, polyprotein processing, and replication provide multiple intervention points, among which proteolytic processing by the main protease represents a particularly vulnerable target. A detailed timeline summarizing the start of the pandemic, the emergence of variants, vaccine rollouts, and key therapeutic milestones during the COVID-19 pandemic is presented in Fig. 1.

## Main protease (Mpro) as a strategic antiviral target

Among the viral proteins essential for SARS-CoV-2 replication, the main protease (Mpro) has emerged as one of the most attractive targets for antiviral drug development. Mpro mediates the proteolytic cleavage of the viral polyproteins pp1a and pp1ab at multiple conserved sites, releasing functional non-structural proteins required for viral replication and transcription (Hoang et al. 2023; Lee et al. 2022). Inhibition of this enzyme therefore disrupts the viral life cycle at an early and critical stage (Zagórska et al. 2024). A key advantage of Mpro as a therapeutic target lies in its high degree of sequence and structural conservation across coronaviruses, including SARS-CoV, MERS-CoV, and circulating SARS-CoV-2 variants (Zhang et al. 2020). This conservation reduces susceptibility to mutation-driven resistance and supports the development of broad-spectrum coronavirus inhibitors. Unlike the spike protein, Mpro is not subject to strong immune selection pressure, making it less prone to antigenic drift. Furthermore, the absence of closely related human homologues minimises the risk of off-target effects, strengthening its suitability as a drug target. Collectively, these features position Mpro as a cornerstone target for sustained antiviral discovery and pandemic preparedness strategies.

**Fig. 2:**
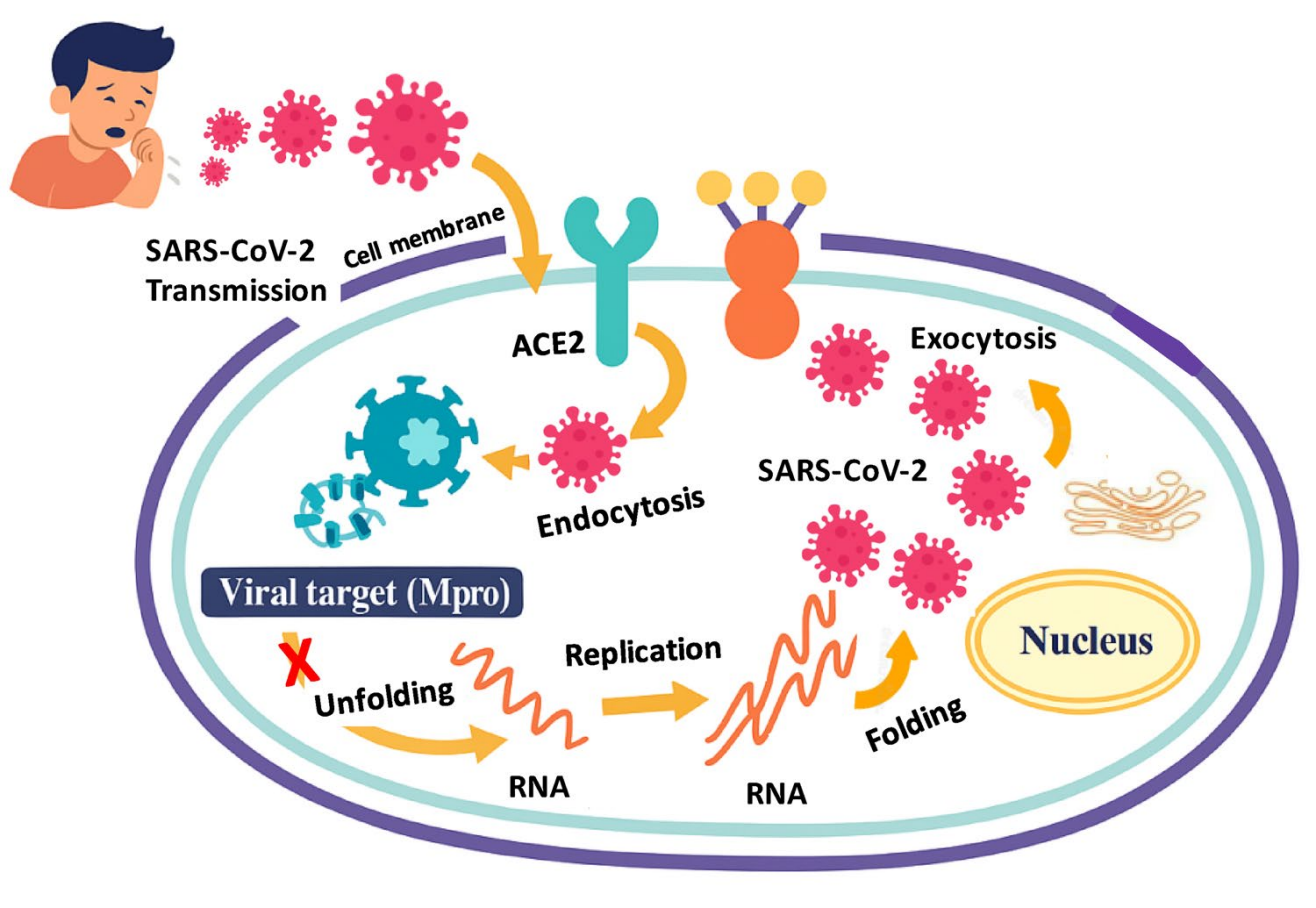
Schematic representation of the SARS-CoV-2 replication cycle and the role of the main protease (Mpro) as a therapeutic target. The figure illustrates the key steps of SARS-CoV-2 infection and replication. The virus binds to the ACE2 receptor on the host cell surface and enters via endocytosis. Once inside, the viral RNA is released and translated into large polyproteins, which are cleaved by the main protease (Mpro) into essential non-structural proteins required for viral replication. Newly assembled viruses are then released from the host cell through exocytosis, continuing the infection cycle. Inhibition of Mpro blocks this cleavage process, thereby halting viral replication and preventing the production of infectious virions

Figure 2 illustrates the viral replication cycle of SARS-CoV-2, highlighting the critical role of the Mpro in processing polyproteins and where Mpro inhibitors can disrupt viral replication.

## Medicinal plants: opportunities and critical challenges

Medicinal plants have long served as a foundational source of therapeutic agents, contributing numerous clinically approved drugs such as quinine, artemisinin and aspirin (Newman and Cragg 2020). Their pharmacological potential arises from a rich diversity of secondary metabolites, including alkaloids, flavonoids, phenolics, terpenoids and glycosides, many of which exhibit antiviral, anti-inflammatory and immunomodulatory properties (Li and Peng 2013). During the early stages of the COVID-19 pandemic, medicinal plants were widely used across many regions as supportive or complementary interventions, particularly in settings with limited access to conventional healthcare (Vellingiri et al. 2020; Yang et al. 2020; Chikowe et al. 2021).

While ethnobotanical knowledge provides valuable starting points for drug discovery, heavy reliance on traditional use alone introduces significant limitations. Ethnomedicinal approaches tend to prioritise a relatively small subset of well-known species, potentially overlooking underexplored taxa with novel chemistry (Richard-Bollans et al. 2023). Furthermore, claims regarding the efficacy of herbal remedies against COVID-19 are often based on anecdotal evidence, in vitro studies, or in silico predictions with limited support from rigorous clinical trials. Variability in plant chemotypes, differences in preparation methods, lack of dose standardisation, potential toxicity at high concentrations and herb-drug interactions further complicate their translation into evidence-based therapeutics. Therefore, medicinal plants should be viewed not as direct substitutes for conventional antivirals, but as a chemically rich discovery space requiring systematic, data-driven exploration. Integrating computational prioritisation, chemical profiling and experimental validation is essential to move beyond descriptive ethnopharmacology toward reproducible and clinically relevant antiviral lead discovery (Pirintsos et al. 2022). Table 1 highlights some medicinal plants used during COVID-19 pandemic with reported pharmacological effects and therapeutic uses.

**Table 1:** Summarises medicinal plants reported during the COVID-19 pandemic, illustrating the chemical and ethnopharmacological space that has informed early computational and experimental investigations rather than confirmed antiviral efficacy

Medicinal plant	Common name	Pharmacological effects	Medicinal uses	Image	References
*Artemisia afra*	African wormwood	Anti-inflammatory Antimicrobial Antioxidant	Coughs Colds Fever		Chebaibi et al. (2022)
*Zingiber officinale*	Ginger	Anti-inflammatory Immune-boosting	Immune support Pain relief Arthritis		Kadhim et al. (2024)
*Allium sativum*	Garlic	Antimicrobial Immune-modulating	Immune system support Cold Fever		Tesfaye (2021)
*Curcuma longa*	Turmeric	Anti-inflammatory Antioxidant	Coughs Asthma Diabetes		Fuloria et al. (2022)
*Sutherlandia frutescens*	Cancer bush	Anti-cancer Anti-inflammatory Anti-diabetic Immunomodulatory	Immune support Stress relief		Ojewole (2004)
*Glycyrrhiza glabra*	Licorice	Antiviral Anti-inflammatory Antiasthmatic	Cough relief Respiratory Circulatory enhancement		Wahab et al. (2021)
*Ocimum gratissimum*	African basil	Antioxidant Anti-inflammatory Antimicrobial Neuroprotective	Respiratory conditions General wellness Fever Diabetes		Ugbogu et al. (2021)

## Machine learning in drug discovery: capabilities and limitations

Machine learning (ML) has emerged as a powerful tool for accelerating early-stage drug discovery by enabling rapid analysis of large chemical and biological datasets In antiviral research, ML models are increasingly applied to predict compound bioactivity, prioritise chemical libraries and guide virtual screening against viral targets such as SARS-CoV-2 Mpro (Paul et al. 2021; Vemula et al. 2023). Supervised learning approaches, including random forest, support vector machines and gradient boosting are commonly used to classify compounds as active or inactive and to predict pharmacokinetic or toxicity-related properties (Cerchia and Lavecchia 2023; Dara et al. 2022; Priya et al. 2022), while unsupervised methods, such as clustering and dimensionality reduction facilitate chemical space exploration and diversity analysis (Chen et al. 2023; Greenacre et al. 2022).

Several recent studies illustrate the practical utility of ML in antiviral discovery against SARS-CoV-2 targets. For example, Ashraf et al. (2023) trained multiple supervised classifiers to distinguish active from inactive SARS-CoV-2 Mpro inhibitors, achieving prediction accuracies exceeding 90%. In parallel, Beck et al. (2020) employed a deep learning-based drug-target interaction model to identify existing antiviral drugs with potential activity against SARS-CoV-2, highlighting the value of ML-driven drug repurposing during emerging health crises. Collectively, these studies demonstrate that ML can substantially accelerate virtual screening and candidate prioritisation when supported by carefully curated training data and appropriate validation strategies.

Despite their promise, ML-driven drug discovery faces several critical challenges. Model performance is highly dependent on the quality, size and diversity of training data, and many SARS-CoV-2 datasets are limited, biased toward known chemotypes, or imbalanced between active and inactive compounds (van Tilborg et al. 2024). Reported high accuracies often reflect internal validation rather than robust external testing, raising concerns about generalisability to novel chemical space. In addition, complex models, particularly deep learning architectures frequently operate as “black boxes”, limiting interpretability and mechanistic insight (Mirza et al. 2019; Ahmed et al. 2024a, b; Rudin 2019). To maximise impact, ML predictions must therefore be interpreted cautiously and integrated with experimental validation. Best practices include the use of external test sets, decoy-aware benchmarking, applicability domain analysis, and explainable AI frameworks (Longo et al. 2024). When embedded within a broader discovery pipeline rather than used in isolation, ML serves as an efficient hypothesis-generation tool that can substantially reduce the experimental search space.

## Metabolomics as an experimental bridge between prediction and chemistry

**Fig. 3:**
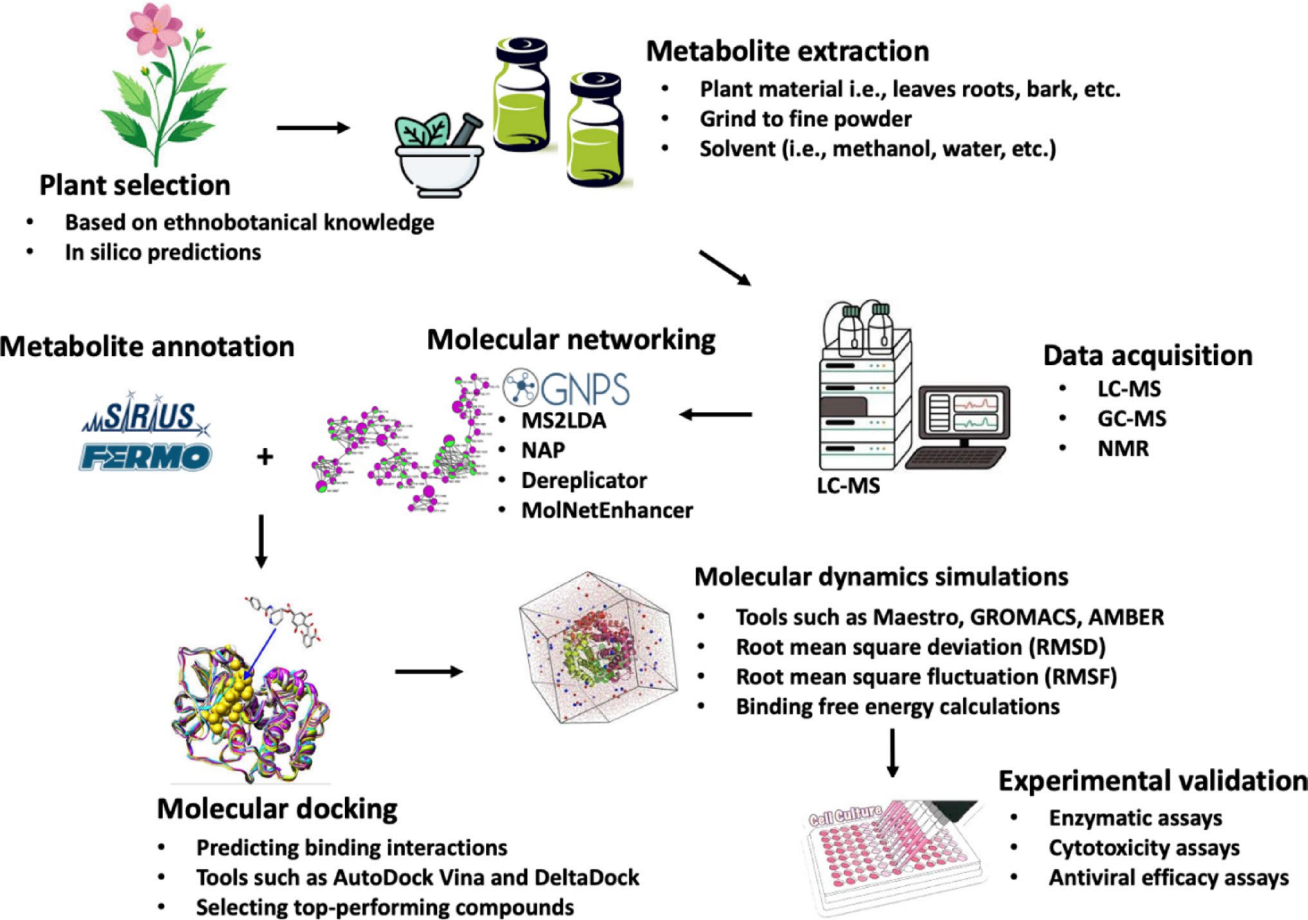
Integrative pipeline for the discovery of bioactive compounds from medicinal plants using metabolomics and computational approaches. The workflow begins with the selection and collection of medicinal plants, followed by extraction of phytochemicals using suitable solvents. Extracts are analysed using liquid chromatography–mass spectrometry (LC–MS), generating complex metabolite profiles. Metabolite annotation and dereplication are enhanced using platforms like GNPS molecular networking and annotation tools such as SURIUS. Putative bioactive compounds are then prioritized for target interaction studies using molecular docking and molecular dynamics simulations. In silico predictions are validated using in vitro assays. This integrative approach accelerates the identification and validation of plant-derived drug candidates

Metabolomics provides a critical experimental link between computational predictions and biochemical reality by enabling comprehensive profiling of small molecules within complex biological matrices. In medicinal plant-based drug discovery, LC–MS-based metabolomics allows confirmation of machine learning-predicted compounds, dereplication of known metabolites, and discovery of previously unreported chemical entities (Guo et al. 2023). Modern metabolomics workflows (Fig. 3) integrate high-resolution LC–MS analysis with computational annotation tools and molecular networking platforms such as GNPS (Azwanida 2015; Alotaibi et al. 2023; Wang et al. 2024). These methods facilitate the organisation of complex spectral data, enable rapid annotation of metabolite families, and guide prioritisation of compounds for downstream molecular docking, molecular dynamics simulations and biological assays (Amorim et al. 2024; Esimbekova et al. 2023).

Numerous studies have demonstrated the utility of LC–MS-based metabolomics for linking chemical profiles to biological activity. For example, Anandika Lestari et al. (2024) applied LC–MS metabolomics with in silico docking to identify and validate antihyperglycemic and antioxidant flavonoids from *Melastoma malabathricum* leaves. Wang et al. (2022) showed how LC–MS-based molecular networking combined with docking can predict bioactive compounds in *Salvia* species, accelerating lead selection against aldose reductase and plasmogen. Similarly, Ahmed et al. (2024a, b) used LC–MS molecular networking to annotate plant metabolites and link these to in vitro activity and cytotoxicity, illustrating an efficient pipeline from profiling to mechanistic insights. These studies highlight how metabolomics not only enables detection of chemically diverse compounds but also connects them to biological activity, supporting early-stage drug discovery decisions.

However, metabolomics itself is not without limitations. Compound annotation remains probabilistic as many LC–MS identifications remain at MSI level 2–3 confidence, isomeric compounds are difficult to distinguish without orthogonal techniques, incomplete spectral libraries can lead to false annotations, and biological relevance must ultimately be confirmed through functional assays. When strategically integrated with computational modelling and experimental validation, metabolomics acts as a powerful filter that enhances confidence in candidate selection and improves the translational relevance of plant-derived drug discovery efforts.

### Network pharmacology: systems-level insight

While metabolomics enables the identification and validation of bioactive compounds from medicinal plants, understanding how these compounds act at a systems level requires complementary approaches. This is where network pharmacology comes in. Network pharmacology provides a systems-level framework for understanding how bioactive compounds interact with multiple molecular targets and biological pathways (Hopkins 2008; Li and Zhang 2013). This approach is particularly relevant for complex diseases such as COVID-19, where viral infection triggers interconnected host immune, inflammatory, and metabolic responses (Zhou et al. 2020a, b). By integrating compound-target predictions with pathway and disease networks, network pharmacology moves beyond the traditional single-target paradigm toward a multitarget and multipathway perspective (Zhang et al. 2019).

Recent studies have successfully demonstrated the power of combining metabolomics and network-based approaches for prioritising plant-derived antivirals against SARS-CoV-2. For instance, Qian and Zeng (2024) integrated UHPLC-MS-based metabolomics with network analysis to reveal that *Jinhua Qinggan Granule* mitigates inflammation by targeting immune-related signalling networks. Similarly, Msobo et al. (2025) employed LC–MS-based metabolomics integrated with network pharmacology to investigate *Salvia* species against COVID-19, identifying metabolites with strong predicted interactions against SARS-CoV-2 targets and highlighting pathways relevant to antiviral activity. Together these studies illustrate how coupling experimental metabolite profiling with computational modelling can strengthen confidence in candidate selection and help bridge the gap between phytochemical complexity and mechanism-oriented antiviral discovery,

Despite these promising efforts, most existing studies employ machine learning, metabolomics, network pharmacology or other computational tools in isolation or connect them only loosely in sequential workflows. This fragmentation leads to gaps in data interpretation, redundancy in compound screening, and inefficiencies in progressing from in silico predictions to experimental validation. The lack of a standardized framework that systematically integrates machine learning-driven prediction, metabolomics-based validation and network analysis limits the pace and precision of plant-based drug discovery. To bridge this gap, a unified computational-experimental pipeline is needed, one that seamlessly connects each stage of discovery from compound prediction to biological validation. Such an approach would not only enable more efficient compound prioritization but also provide a deeper understanding of how predicted metabolites act within biological systems. Building upon this foundation, the following section proposes a stepwise unified workflow that integrates machine learning, enrichment analysis, metabolomics, molecular docking, molecular dynamics and experimental bioassays to accelerate the identification and validation of antiviral compounds from medicinal plants.

## Toward a unified computational-experimental pipeline for medicinal plant-based drug discovery

**Fig. 4:**
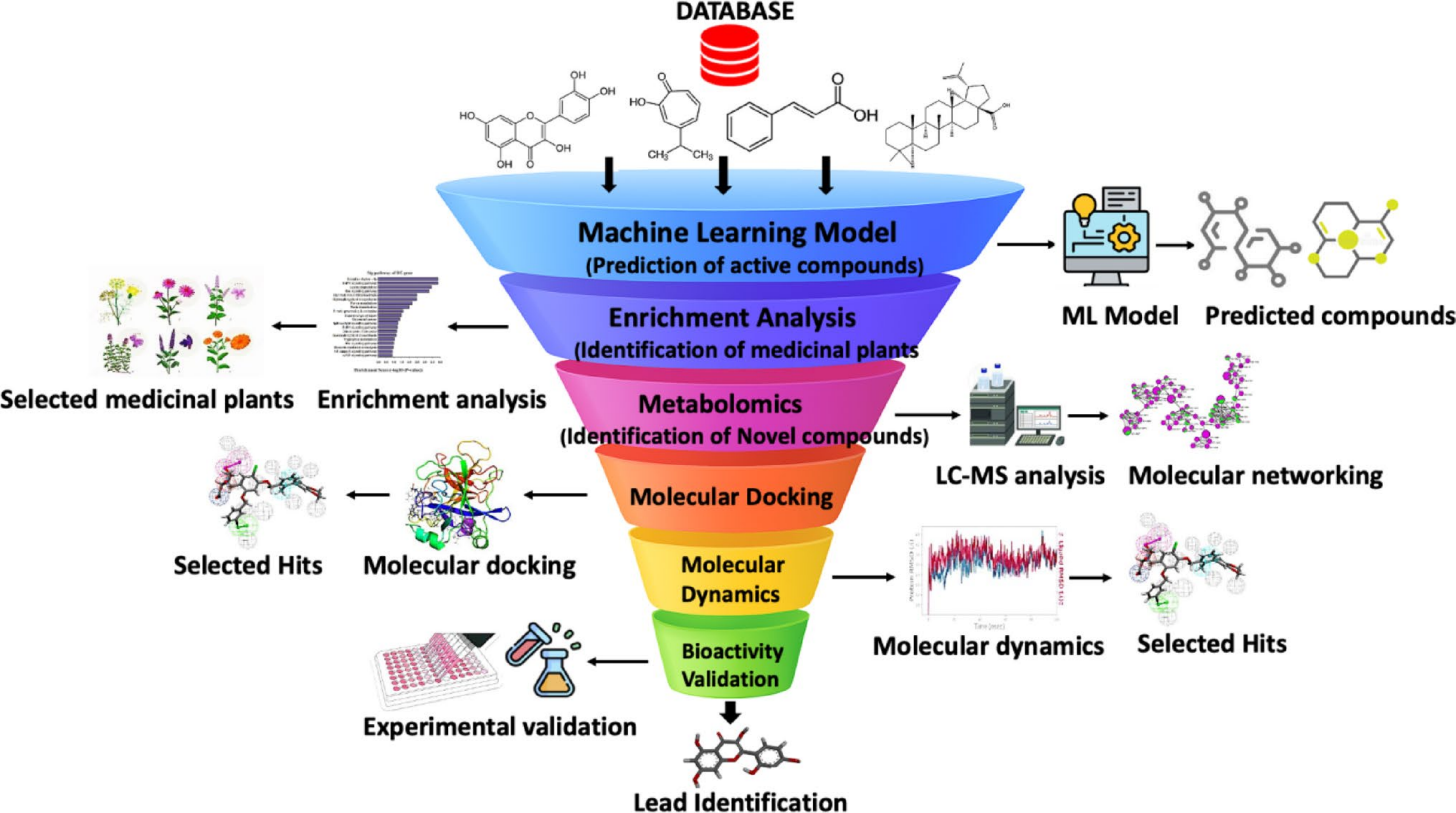
Integrative pipeline combining machine learning, metabolomics, and molecular modelling for bioactive compound discovery. The workflow begins with a machine learning model trained on chemical structure–activity data to predict potential bioactive compounds. Enrichment analysis is then used to identify medicinal plant sources likely to contain these compounds. Metabolomics, particularly LC–MS-based analysis, enables the identification of novel plant metabolites. Candidate compounds are subjected to molecular docking and molecular dynamics simulations to evaluate target binding affinity and interaction stability. Finally, experimental bioassays are performed to validate biological activity, completing the pipeline from computational prediction to empirical validation

Plant-based antiviral discovery has traditionally relied on ethnobotanical selection followed by phytochemical isolation and biological testing, often progressing in a linear and resource-intensive manner. More recent approaches frequently conclude at a level of in silico screening, creating a disconnect between computational predictions and experimental validation. To address these limitations, a unified computational-experimental pipeline is proposed that integrates machine learning, enrichment analysis, metabolomics, molecular modelling and biological assays into a coherent and iterative workflow. The pipeline (Fig. 4) initiates with machine learning models trained on curated structure–activity datasets (e.g., ChEMBL, BindingDB, or SARS-CoV-2 screening datasets) to predict compounds with potential inhibitory activity against SARS-CoV-2 Mpro. Rather than treating these predictions as final outputs, predicted active compounds are traced back to their botanical sources using enrichment analysis. This step enables data-driven prioritisation of medicinal plant species statistically enriched in predicted actives, expanding discovery beyond traditionally studied taxa and reducing bias introduced by ethnobotanical familiarity alone.

Prioritised plant species are then subjected to LC–MS-based metabolomic profiling to generate comprehensive chemical fingerprints. Metabolomics serves as a critical validation layer by confirming the presence of predicted compounds, dereplicating known metabolites and revealing structurally novel entities absent from training datasets. Annotated metabolites are subsequently evaluated using molecular docking and molecular dynamics simulations to assess binding modes, interaction stability, and dynamic behaviour within the Mpro active or allosteric sites. Candidate compounds that demonstrate favourable computational profiles are advanced to experimental validation, including enzyme inhibition assays, cytotoxicity testing, and where applicable, kinetic characterisation. Network pharmacology may be incorporated at this stage to contextualise validated compounds within host-virus interaction networks and to explore potential multitarget effects. Importantly, this framework is iterative, with experimental results informing model refinement and future predictions. By unifying predictive modelling, chemical verification, and biological validation, this pipeline (Fig. 4) provides a scalable and reproducible strategy for translating medicinal plant chemistry into credible antiviral leads.

### Challenges and translational considerations

Despite their promise, integrated computational-experimental approaches face several translational challenges. Machine learning models are vulnerable to data bias, limited chemical diversity, and overfitting, particularly when trained on small or imbalanced antiviral datasets. In metabolomics, incomplete spectral libraries, batch effects, and probabilistic annotations complicate metabolite identification and cross-study reproducibility. Moreover, in vitro antiviral activity does not always translate to in vivo efficacy or clinical relevance due to pharmacokinetic and toxicity constraints. Addressing these challenges through rigorous validation and transparent reporting is essential for successful translation.

## Conclusion and future perspectives

The COVID-19 pandemic exposed critical limitations in conventional antiviral drug discovery, particularly the difficulty of rapidly and systematically exploiting the chemical diversity of natural products. Although vaccines and repurposed antivirals have substantially reduced disease severity, the continued circulation of SARS-CoV-2 and the emergence of new variants underscore the importance of sustained discovery efforts targeting conserved viral proteins such as the main protease (Mpro). This review highlights medicinal plants as a valuable yet underutilised source of antiviral chemical diversity, while emphasising the limitations of ethnopharmacology-driven and purely in silico approaches. By critically integrating machine learning, LC–MS-based metabolomics, molecular modelling, and network pharmacology, we present a unified computational-experimental framework that strengthens compound prioritisation, reduces false positives and bridges the gap between prediction and biological relevance.

Future progress will depend on the availability of high-quality, standardised datasets, improved model interpretability, and rigorous experimental validation. Equally important are ethical considerations, including sustainable plant sourcing and responsible engagement with indigenous knowledge systems. When applied judiciously, the integrative pipeline outlined here offers a robust and adaptable model for antiviral discovery that extends beyond COVID-19, supporting preparedness for future viral threats while advancing responsible innovation in natural product research.

## Data Availability

No datasets were generated or analysed during the current study.
